# Supplementary material for: Unsupervised clustering of temporal patterns in high-dimensional neuronal ensembles using a novel dissimilarity measure
Source: PLoS Comput Biol. 2018 Jul 6;14(7):e1006283. doi: 10.1371/journal.pcbi.1006283 (PMC6051652; doi:10.1371/journal.pcbi.1006283)
Supplement: S1 Fig — For each epoch (1), the cross-correlation is computed for each pair of neurons (2). These cross-correlations are then normalized to unit mass (3). For each pair of epochs and pair of neurons (4) we then compute the EMD. The EMDs are then averaged over all eligible neuron pairs (i.e. pairs of neurons active in both epoch k and m) to compute the SPOTDis between epochs k and m. (PDF) [file pcbi.1006283.s001.pdf]

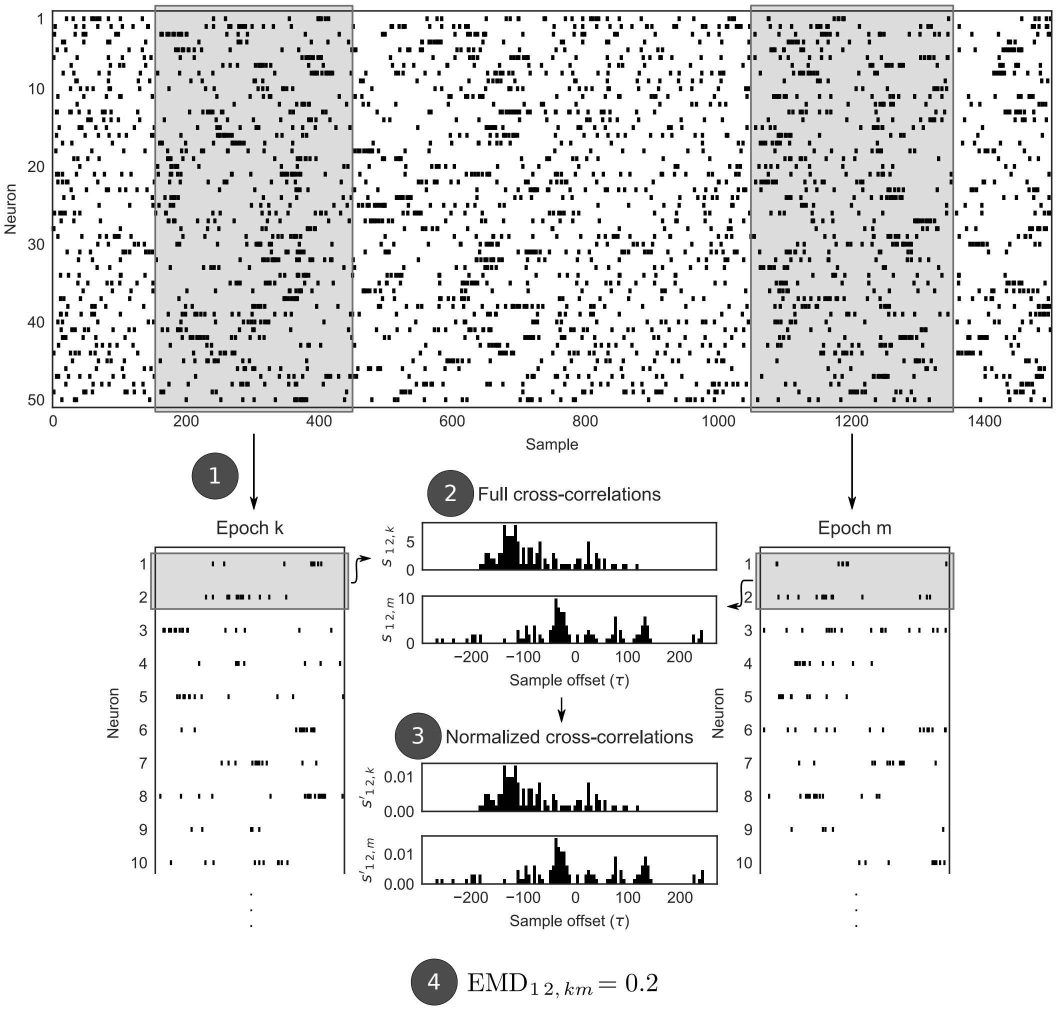

Figure S1: Illustration of the SPOTDis computation. For each epoch (1), the cross-correlation is computed for each pair of neurons (2). These cross-correlations are then normalized to unit mass (3). For each pair of epochs and pair of neurons (4) we then compute the EMD. The EMDs are then averaged over all eligible neuron pairs (i.e. pairs of neurons active in both epoch k and m) to compute the SPOTDis between epochs k and m.
