# Supplementary material for: Unsupervised clustering of temporal patterns in high-dimensional neuronal ensembles using a novel dissimilarity measure
Source: PLoS Comput Biol. 2018 Jul 6;14(7):e1006283. doi: 10.1371/journal.pcbi.1006283 (PMC6051652; doi:10.1371/journal.pcbi.1006283)
Supplement: S2 Fig — Showing the first 5 iterations of the EMD algorithm on simple example cross correlation histograms. Each normalized cross-correlation (top and bottom) belongs to one epoch. In each step the mass is transported from the top epoch to the highlighted parts of the histogram and the histogram entries between which mass is transported are connected. (PDF) [file pcbi.1006283.s002.pdf]

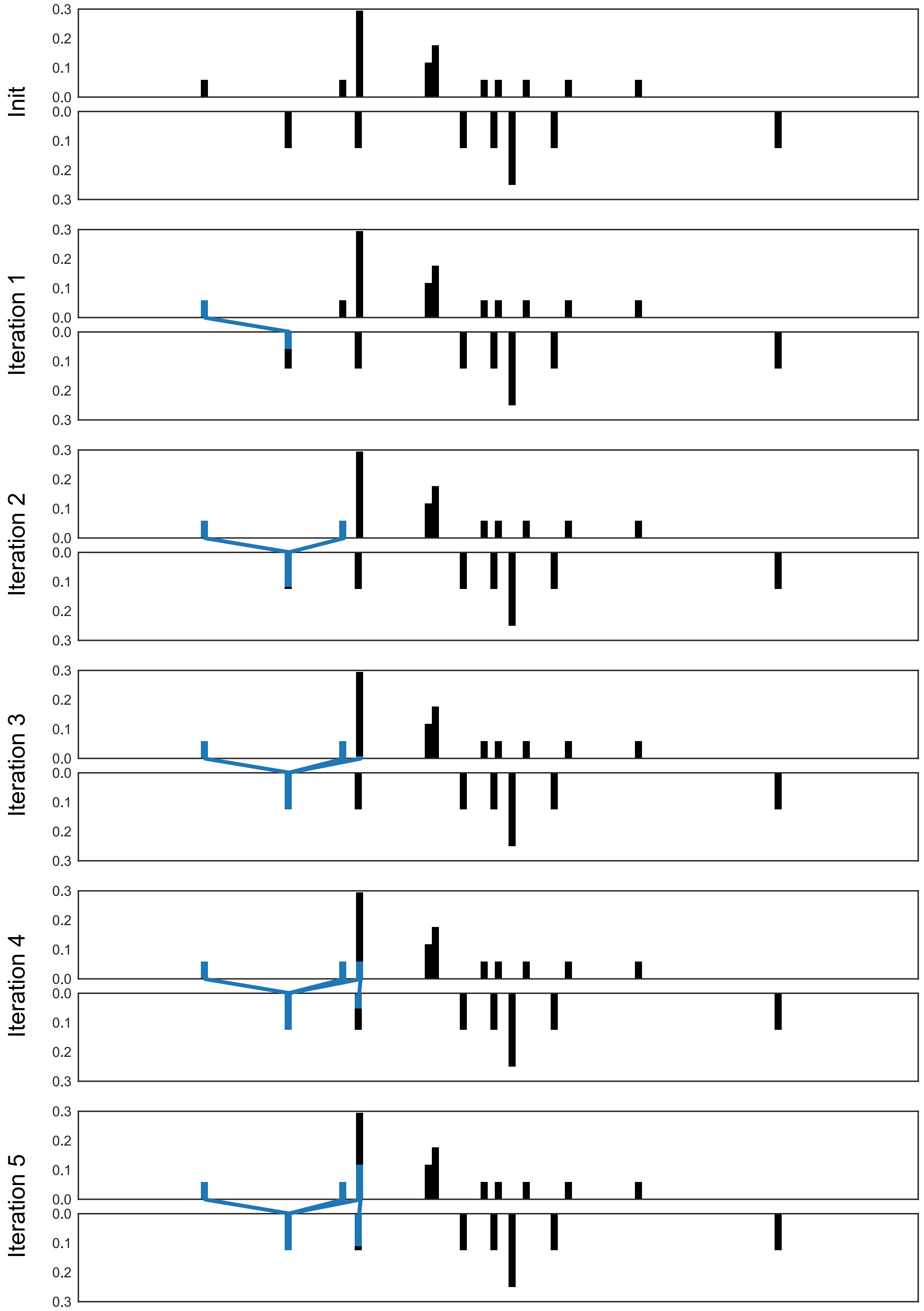

Figure S2: Illustration of the EMD computation. Showing the first 5 iterations of the EMD algorithm on simple example cross correlation histograms. Each normalized cross-correlation (top and bottom) belongs to one epoch. In each step the mass is transported from the top epoch to the highlighted parts of the histogram and the histogram entries between which mass is transported are connected.
