# Supplementary material for: Unsupervised clustering of temporal patterns in high-dimensional neuronal ensembles using a novel dissimilarity measure
Source: PLoS Comput Biol. 2018 Jul 6;14(7):e1006283. doi: 10.1371/journal.pcbi.1006283 (PMC6051652; doi:10.1371/journal.pcbi.1006283)
Supplement: S3 Fig — Simulation parameters were λin = 0.35 spks/sample, λout = 0.05 spks/sample, Tepoch = 300 samples, Tpulse = 30 samples. Homogeneous noise was generated according to a homogeneous Poisson process, while each patterned noise epoch was an instantiation of a unique pattern, that was randomly generated with the same statistics as the four recurring patterns (i.e. for each neuron it had the same values of the pulse duration, λin and λout). The t-SNE embedding shows that homogeneous noise forms a separate cluster, while patterned noise does not. (PDF) [file pcbi.1006283.s003.pdf]

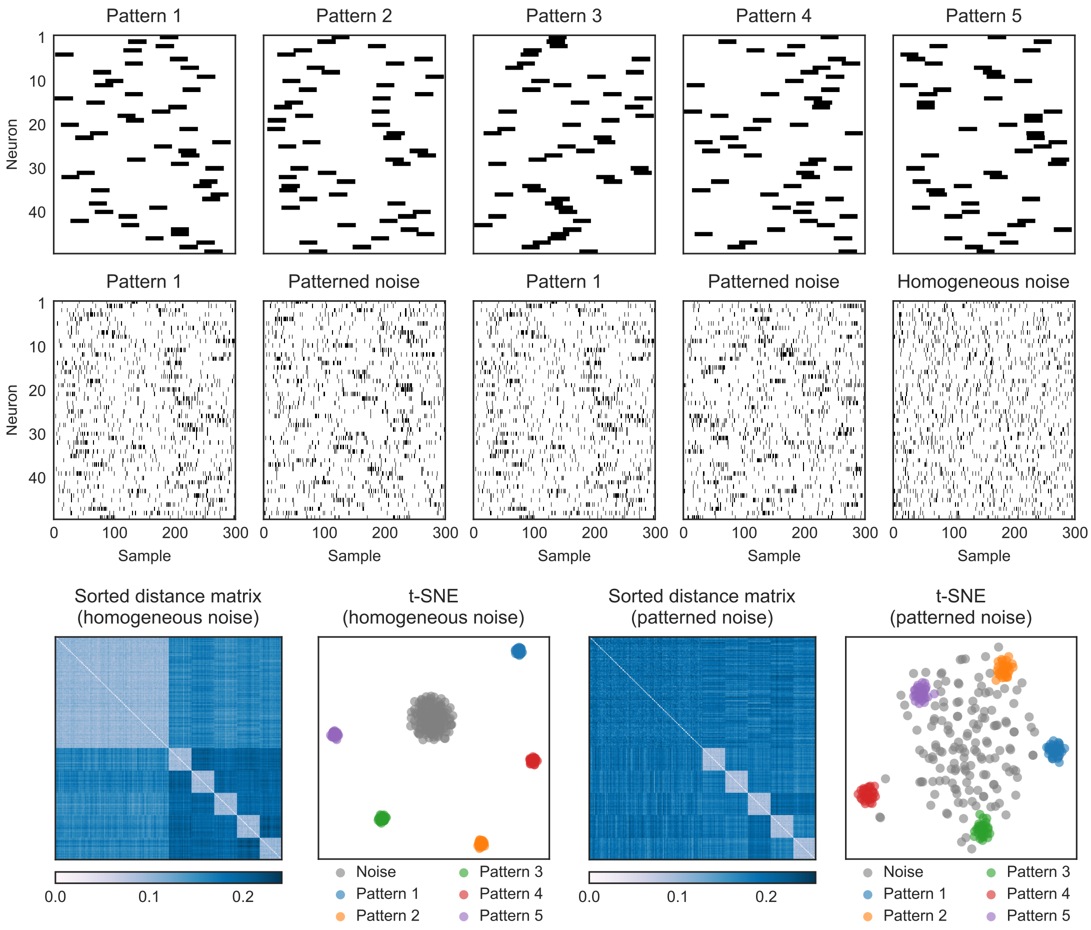

Figure S3: Example figure to illustrate the difference between homogeneous noise and patterned noise. Simulation parameters were $\lambda_{in} = 0.35$ spks/sample, $\lambda_{out} = 0.05$ spks/sample, $T_{epoch} = 300$ samples, $T_{pulse} = 30$ samples. Homogeneous noise was generated according to a homogeneous Poisson process, while each patterned noise epoch was an instantiation of a unique pattern, that was randomly generated with the same statistics as the four recurring patterns (i.e. for each neuron it had the same values of the pulse duration, λ_{in} and λ_{out}). The t-SNE embedding shows that homogeneous noise forms a separate cluster, while patterned noise does not.
