# Supplementary material for: Unsupervised clustering of temporal patterns in high-dimensional neuronal ensembles using a novel dissimilarity measure
Source: PLoS Comput Biol. 2018 Jul 6;14(7):e1006283. doi: 10.1371/journal.pcbi.1006283 (PMC6051652; doi:10.1371/journal.pcbi.1006283)
Supplement: S4 Fig — Shown are the excess of mass (EOM) and the leaf cluster selection methods of the HDBSCAN algorithm (see Methods). Grey points are identified as noise points by HDBSCAN, other colors correspond to clusters. (PDF) [file pcbi.1006283.s004.pdf]

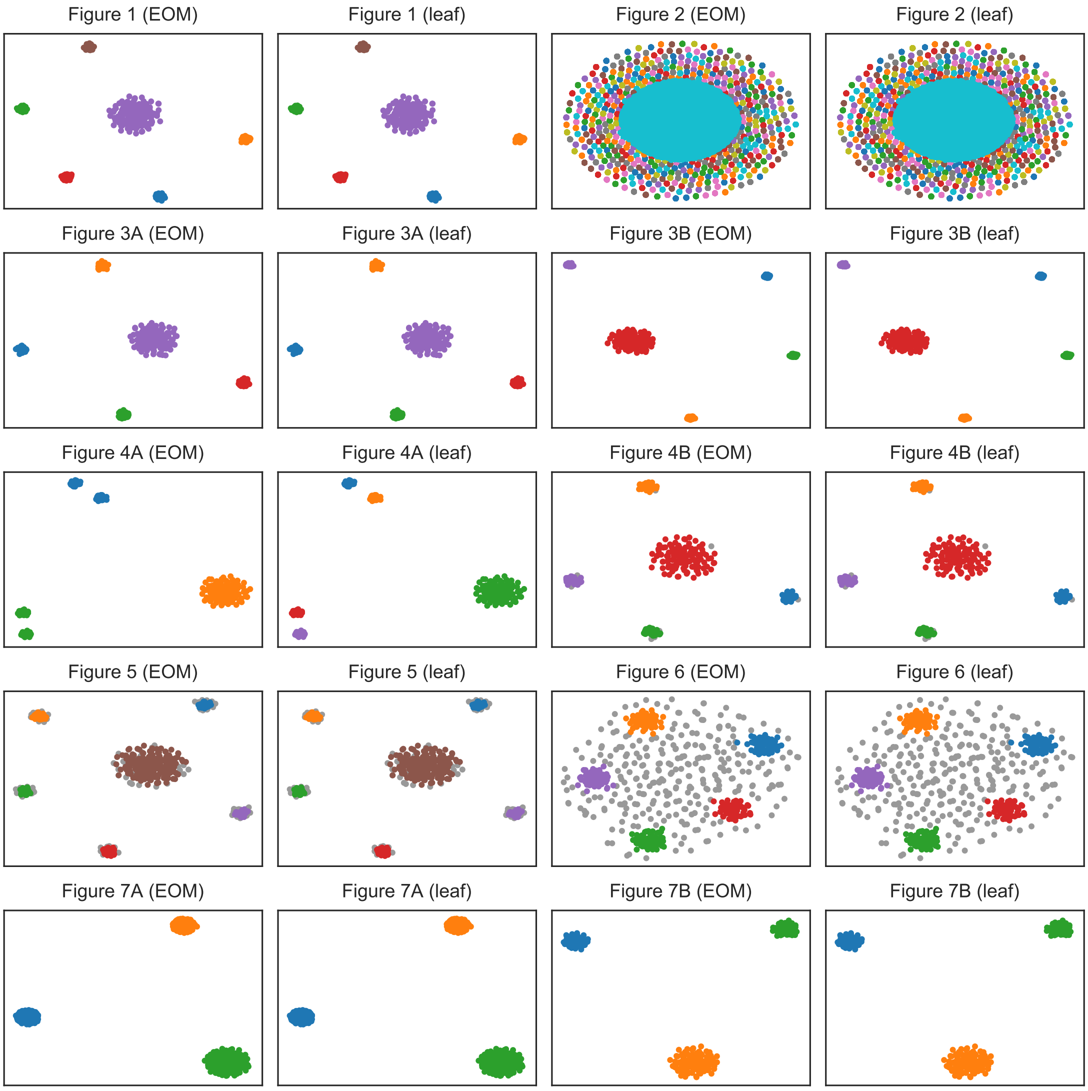

Figure S4: HDBSCAN labels for each of the seven main figures. Shown are the excess of mass (EOM) and the leaf cluster selection methods of the HDBSCAN algorithm (see Methods). Grey points are identified as noise points by HDBSCAN, other colors correspond to clusters.
