# Supplementary material for: Unsupervised clustering of temporal patterns in high-dimensional neuronal ensembles using a novel dissimilarity measure
Source: PLoS Comput Biol. 2018 Jul 6;14(7):e1006283. doi: 10.1371/journal.pcbi.1006283 (PMC6051652; doi:10.1371/journal.pcbi.1006283)
Supplement: S5 Fig — Same settings as in Fig 1, but now using 750 noise epochs and only 30 epochs per cluster. Even though there is a very large number of noise epochs, t-SNE embedding is still able to reveal the separate clusters. (PDF) [file pcbi.1006283.s005.pdf]

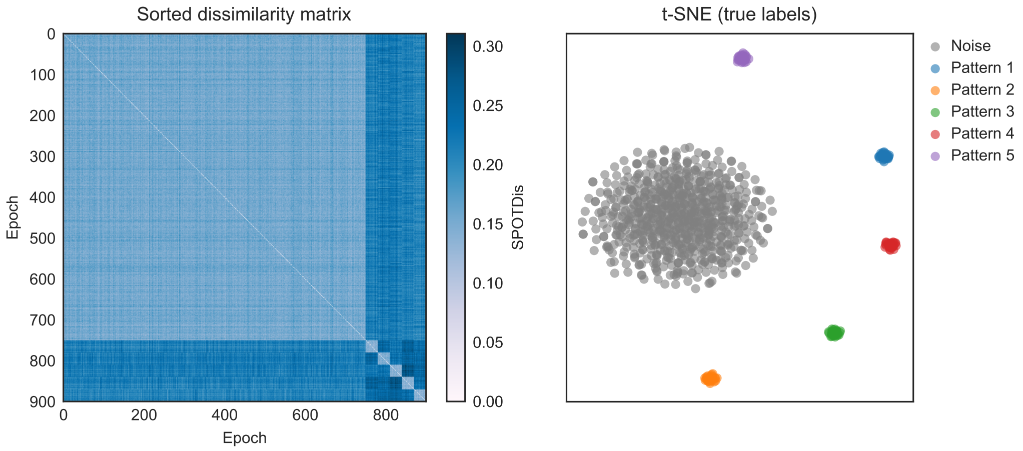

Figure S5: SPOTDisClust performance for cases where there are many noise epochs and few cluster epochs. Same settings as in Figure 1, but now using 750 noise epochs and only 30 epochs per cluster. Even though there is a very large number of noise epochs, t-SNE embedding is still able to reveal the separate clusters.
