# Supplementary material for: Unsupervised clustering of temporal patterns in high-dimensional neuronal ensembles using a novel dissimilarity measure
Source: PLoS Comput Biol. 2018 Jul 6;14(7):e1006283. doi: 10.1371/journal.pcbi.1006283 (PMC6051652; doi:10.1371/journal.pcbi.1006283)
Supplement: S6 Fig — We consider four rate modulations (top left) each consisting of a single activation pulse and a baseline. We then generated spikes from the four rate modulations according to an inhomogeneous Poisson process (top right). The rate modulations may represent cross-correlations for neuron pairs or activation patterns for single neurons. We then varied the spike rate inside the activation period and outside the activation period, thereby changing the ratio of spikes inside the pulse period to the amount of spikes outside the pulse period (changing the SNR). We then computed the EMD between the normalized mass of rate modulation k and the spike realization of rate modulation 1, for all k = 1, …, 4, defined as Δk. Here the pattern mass can be interpreted as an average of many spike train realizations of that rate modulation. The figure on the bottom left shows, across many spike train realizations of rate modulation 1, ΔEMD(k) = Δk − Δ1. The dashed lines indicate the EMD between the different patterns. Thus, a value of ΔEMD(k) > 0 indicates that the spike train has a lower EMD to its own corresponding rate modulation than to another rate modulation, i.e. it indicates that the assignment of spike train to rate modulation based on the EMD would be correct. We find that as long as the spike count SNR—which we define as the ratio of spikes inside the pulse period to the amount of spikes outside the pulse period—exceeds 1, the minimum value of Δk occurs when k = 1, that is when the EMD is computed between the realization and its corresponding rate modulation (S6 Fig). Even when the SNR drops significantly below 1, this holds true. Thus, the proximity of a rate modulation to its realization, in terms of EMD, is highly robust to the insertion of noise spikes around the activation pulse period. We find that the same principle holds approximately true when comparing the EMD between two spike train realizations from the same rate modulation vs. spike train realizations from other ra [file pcbi.1006283.s006.pdf]

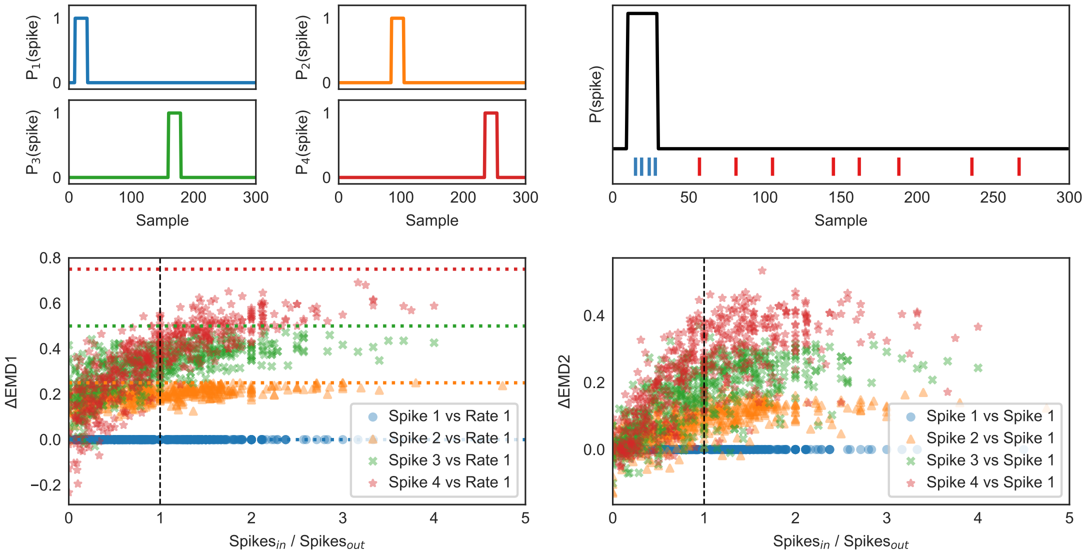

Figure S6: Illustration of how the EMD is a noise-robust measure of distance for spikes. We consider four rate modulations (top left) each consisting of a single activation pulse and a baseline. We then generated spikes from the four rate modulations according to an inhomogeneous Poisson process (top right). The rate modulations may represent cross-correlations for neuron pairs or activation patterns for single neurons. We then varied the spike rate inside the activation period and outside the activation period, thereby changing the ratio of spikes inside the pulse period to the amount of spikes outside the pulse period (changing the SNR). We then computed the EMD between the normalized mass of rate modulation k and the spike realization of rate modulation 1, for all $k = 1, \ldots, 4$, defined as Δ_k. Here the pattern mass can be interpreted as an average of many spike train realizations of that rate modulation. The figure on the bottom left shows, across many spike train realizations of rate modulation 1, $\Delta\text{EMD}(k) = \Delta_k - \Delta_1$. The dashed lines indicate the EMD between the different patterns. Thus, a value of $\Delta\text{EMD}(k) > 0$ indicates that the spike train has a lower EMD to its own corresponding rate modulation than to another rate modulation, i.e. it indicates that the assignment of spike train to rate modulation based on the EMD would be correct. We find that as long as the spike count SNR - which we define as the ratio of spikes inside the pulse period to the amount of spikes outside the pulse period - exceeds 1, the minimum value of Δ_k occurs when $k = 1$, that is when the EMD is computed between the realization and its corresponding rate modulation. Even when the SNR drops significantly below 1, this holds true. Thus, the proximity of a rate modulation to its realization, in terms of EMD, is highly robust to the insertion of noise spikes around the activation pulse period. We find that the same principle holds approximately true when comparing the EMD between two spike train realizations from the same rate modulation vs. spike train realizations from other rate modulation (bottom right). The robustness to insertion of spikes around the activation period can be intuitively understood from the behavior of the EMD transport distance: When comparing e.g. a realization of rate modulation 1 with the mass of pattern 2, we need to move points out of the pulse period of rate modulation 1, move points into the activation period of rate modulation 2, as well as spreading out spike energy to minimize the distance to the baseline. In contrast, when comparing spikes to the same rate modulation, the transport cost only consists of moving a few points in or out of the pulse period, and spreading the points around to minimize the distance to the baseline.
