# Supplementary material for: Unsupervised clustering of temporal patterns in high-dimensional neuronal ensembles using a novel dissimilarity measure
Source: PLoS Comput Biol. 2018 Jul 6;14(7):e1006283. doi: 10.1371/journal.pcbi.1006283 (PMC6051652; doi:10.1371/journal.pcbi.1006283)
Supplement: S7 Fig — (A) Five patterns were generated for a total of 330 neurons, with λin = 0.15 spks/sample, λout = 0.01 spks/sample. Each group of 11 subsequent neurons was assigned to one “virtual electrode”. From this virtual electrode, we then only “recorded” one neuron. The activity of each recorded neuron was then contaminated by randomly inserting spikes from the other “hidden’ neurons. Both the number of contaminating “hidden’ neurons and the fraction of contaminating spikes, as compared to the total number of spikes of each “hidden” neuron, was varied. For example, a value of 5 contaminating neurons and 50% contaminating spikes indicated that 50% of spikes from each of the 5 contaminating “hidden” neurons was added to the recorded neuron. On the left and middle, an original realization of a spike pattern and the observed spike trains after contamination (10 contaminating neurons and each “hidden” neuron contaminating with 100% of its spikes). The table on the right shows the clustering performance compared with the ground-truth (ARI) as a function of spike contamination parameters. B) Patterns were defined using the same firing statistics as in Fig 1. Each group of 5 subsequent neurons was assigned to a “virtual electrode”. The output of all neurons was observed in this case. For each epoch, we then randomly exchanged spikes among the 5 neurons. A contamination probability of 0.25 meant that each spike had a probability of 0.25 to be transferred to another randomly chosen neuron, i.e. inserted into the spike train of that other neuron. On the left and middle are respectively shown the original spike train output, and the resulting spike train pattern after contamination. On the right is shown the HDBSCAN clustering performance compared to ground-truth (ARI) with standard deviations (across 5 repetitions of the simulation). (PDF) [file pcbi.1006283.s007.pdf]

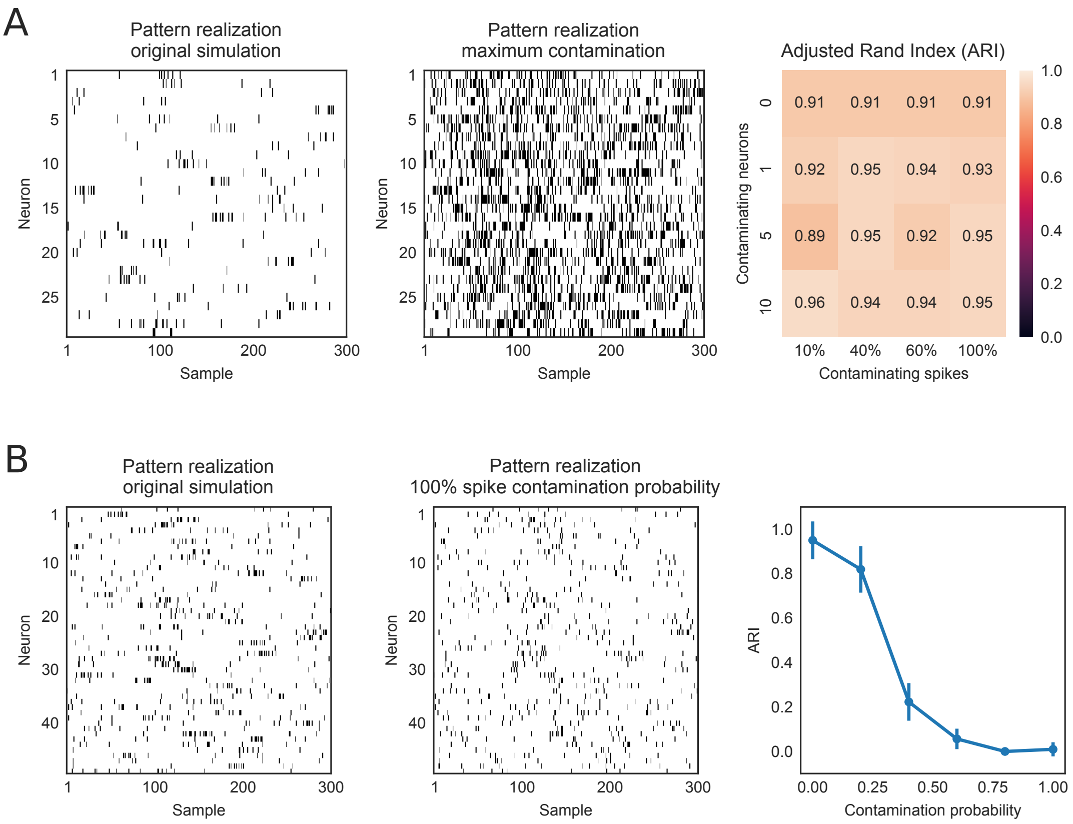

Figure S7: Dependence of clustering performance on spike sorting contamination. (A) Five patterns were generated for a total of 330 neurons, with λ_{in} = 0.15 spks/sample, λ_{out} = 0.01 spks/sample. Each group of 11 subsequent neurons was assigned to one "virtual electrode". From this virtual electrode, we then only "recorded" one neuron. The activity of each recorded neuron was then contaminated by randomly inserting spikes from the other "hidden' neurons. Both the number of contaminating "hidden' neurons and the fraction of contaminating spikes, as compared to the total number of spikes of each "hidden" neuron, was varied. For example, a value of 5 contaminating neurons and 50% contaminating spikes indicated that 50% of spikes from each of the 5 contaminating "hidden" neurons was added to the recorded neuron. On the left and middle, an original realization of a spike pattern and the observed spike trains after contamination (10 contaminating neurons and each "hidden" neuron contaminating with 100% of its spikes). The table on the right shows the clustering performance compared with the ground-truth (ARI) as a function of spike contamination parameters. B) Patterns were defined using the same firing statistics as in Figure 1. Each group of 5 subsequent neurons was assigned to a "virtual electrode". The output of all neurons was observed in this case. For each epoch, we then randomly exchanged spikes among the 5 neurons. A contamination probability of 0.25 meant that each spike had a probability of 0.25 to be transferred to another randomly chosen neuron, i.e. inserted into the spike train of that other neuron. On the left and middle are respectively shown the original spike train output, and the resulting spike train pattern after contamination. On the right is shown the HDBSCAN clustering performance compared to ground-truth (ARI) with standard deviations (across 5 repetitions of the simulation).
