# Supplementary material for: Unsupervised clustering of temporal patterns in high-dimensional neuronal ensembles using a novel dissimilarity measure
Source: PLoS Comput Biol. 2018 Jul 6;14(7):e1006283. doi: 10.1371/journal.pcbi.1006283 (PMC6051652; doi:10.1371/journal.pcbi.1006283)
Supplement: S8 Fig — The simulations are based on the same firing statistics reported for Fig 1. Neurons were divided into 10 groups of subsequent 5 neurons, each constituting a “virtual electrode”. Whenever two neurons fired simultaneously (i.e. at the same sample) within the group of 5, we removed the two spikes with a certain collision probability. The spike rasters on the left and middle panel show, respectively, an original spike train realization from a pattern and the spike train realization after removing the colliding spikes with 100% collision probability. The panel on the right shows HDBSCAN clustering performance compared to ground-truth (ARI) as a function of collision probability, with standard deviations. Same as (A), but now all neurons within a group of 5 fire according to identical patterns, which strongly increases the amount of collisions, and deteriorates clustering performance as collision probability increases. (PDF) [file pcbi.1006283.s008.pdf]

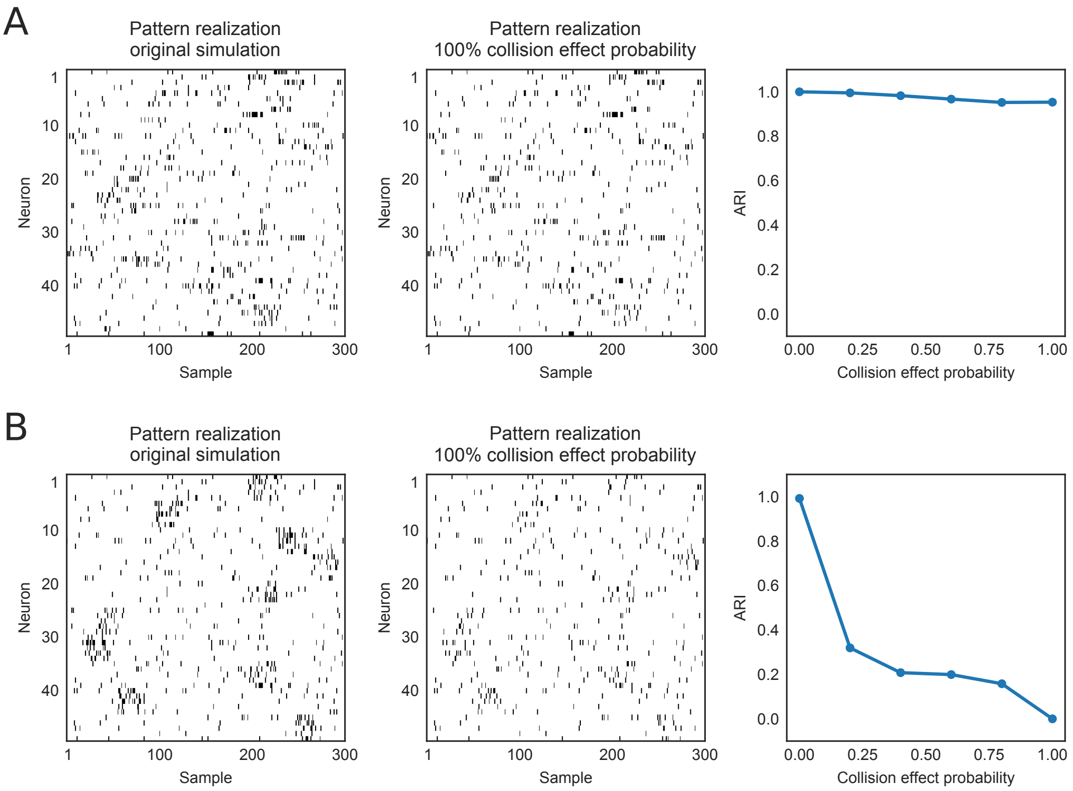

Figure S8: Dependence of clustering performance on spike sorting errors resulting from collisions. The simulations are based on the same firing statistics reported for Figure 1. Neurons were divided into 10 groups of subsequent 5 neurons, each constituting a "virtual electrode". Whenever two neurons fired simultaneously (i.e. at the same sample) within the group of 5, we removed the two spikes with a certain collision probability. The spike rasters on the left and middle panel show, respectively, an original spike train realization from a pattern and the spike train realization after removing the colliding spikes with 100% collision probability. The panel on the right shows HDBSCAN clustering performance compared to ground-truth (ARI) as a function of collision probability, with standard deviations. Same as (A), but now all neurons within a group of 5 fire according to identical patterns, which strongly increases the amount of collisions, and deteriorates clustering performance as collision probability increases.
