# Supplementary material for: Unsupervised clustering of temporal patterns in high-dimensional neuronal ensembles using a novel dissimilarity measure
Source: PLoS Comput Biol. 2018 Jul 6;14(7):e1006283. doi: 10.1371/journal.pcbi.1006283 (PMC6051652; doi:10.1371/journal.pcbi.1006283)
Supplement: S9 Fig — Five patterns were defined by selecting for each pattern a random time point at which the neuron would fire a spike. In addition, we inserted a varying number of noise spikes per neuron according to a homogeneous Poisson process, and also varied the amount of temporal jitter in the pattern spikes. The left panel shows an example for one such pattern, where the pattern spikes are displayed in black and the noise spikes are colored gray. The right panel shows the HDBSCAN cluster quality as compared to ground truth (ARI) with a varying number of noise spikes along with applying an increasing amount of temporal jitter to the pattern spikes. The maximum jitter denoted on the x-axis is defined as a percentage of the total interval length, from which a perturbation value is chosen with uniform probability, for each pattern spike individually. This means that a maximum jitter of 100% corresponds to a jitter chosen uniformly from [−T, T], and 20% corresponds to a jitter uniformly chosen from [−0.2T, 0.2T]. Spikes that are perturbed such that they fall outside of the epoch’s time interval are still considered to be part of the epoch. (PDF) [file pcbi.1006283.s009.pdf]

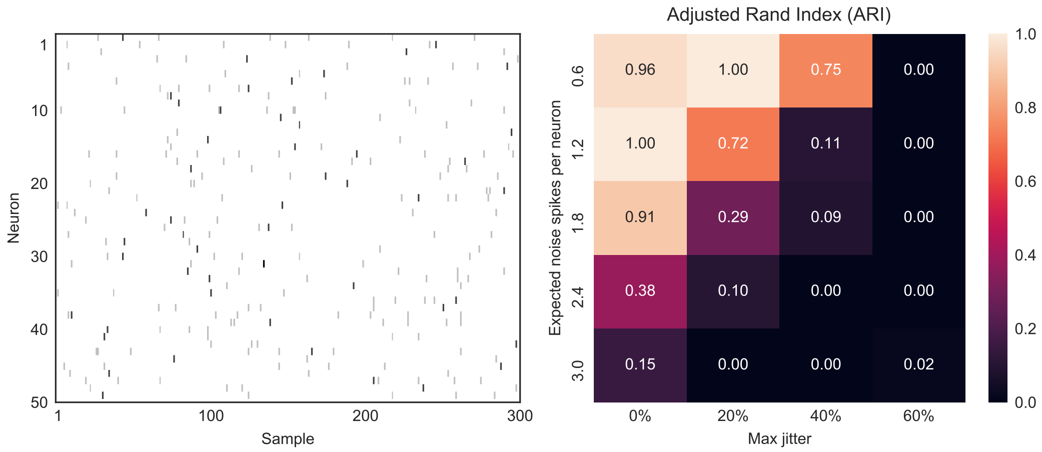

Figure S9: Clustering performance for a case with precise spike sequences. Five patterns were defined by selecting for each pattern a random time point at which the neuron would fire a spike. In addition, we inserted a varying number of noise spikes per neuron according to a homogeneous Poisson process, and also varied the amount of temporal jitter in the pattern spikes. The left panel shows an example for one such pattern, where the pattern spikes are displayed in black and the noise spikes are colored gray. The right panel shows the HDBSCAN cluster quality as compared to ground truth (ARI) with a varying number of noise spikes along with applying an increasing amount of temporal jitter to the pattern spikes. The maximum jitter denoted on the x-axis is defined as a percentage of the total interval length, from which a perturbation value is chosen with uniform probability, for each pattern spike individually. This means that a maximum jitter of 100% corresponds to a jitter chosen uniformly from $[-T, T]$, and 20% corresponds to a jitter uniformly chosen from $[-0.2T, 0.2T]$. Spikes that are perturbed such that they fall outside of the epoch's time interval are still considered to be part of the epoch.
