# Supplementary material for: Unsupervised clustering of temporal patterns in high-dimensional neuronal ensembles using a novel dissimilarity measure
Source: PLoS Comput Biol. 2018 Jul 6;14(7):e1006283. doi: 10.1371/journal.pcbi.1006283 (PMC6051652; doi:10.1371/journal.pcbi.1006283)
Supplement: S10 Fig — For each multi-unit, we computed the spike count in the same temporal window as used for the SPOTDisClust clustering, denoted rik (epoch k, unit i). In (A), we then constructed a normalized population vector as rik′=rik/∑irik for each multi-unit i. We then constructed all pairwise distances between epochs k and m as the L1-norm among these normalized population vectors, Dkmrate=1N∑i=1N|rik′-rim′|. Based on these pairwise distances, we then performed low dimensional t-SNE embedding and HDBSCAN clustering. Shown are the distance matrix, as well as the t-SNE with true labels and cluster labels. The clustering and low-dimensional embedding is unable to separate out all four stimulus directions. In (B), we defined the pairwise distances as the L1-norm on the absolute spike counts, i.e. Dkmrate=1N∑i=1N|rik-rim|, and then performed t-SNE and HDBSCAN clustering. Like in (A), this clustering procedure fails to separate all four stimulus directions. (PDF) [file pcbi.1006283.s010.pdf]

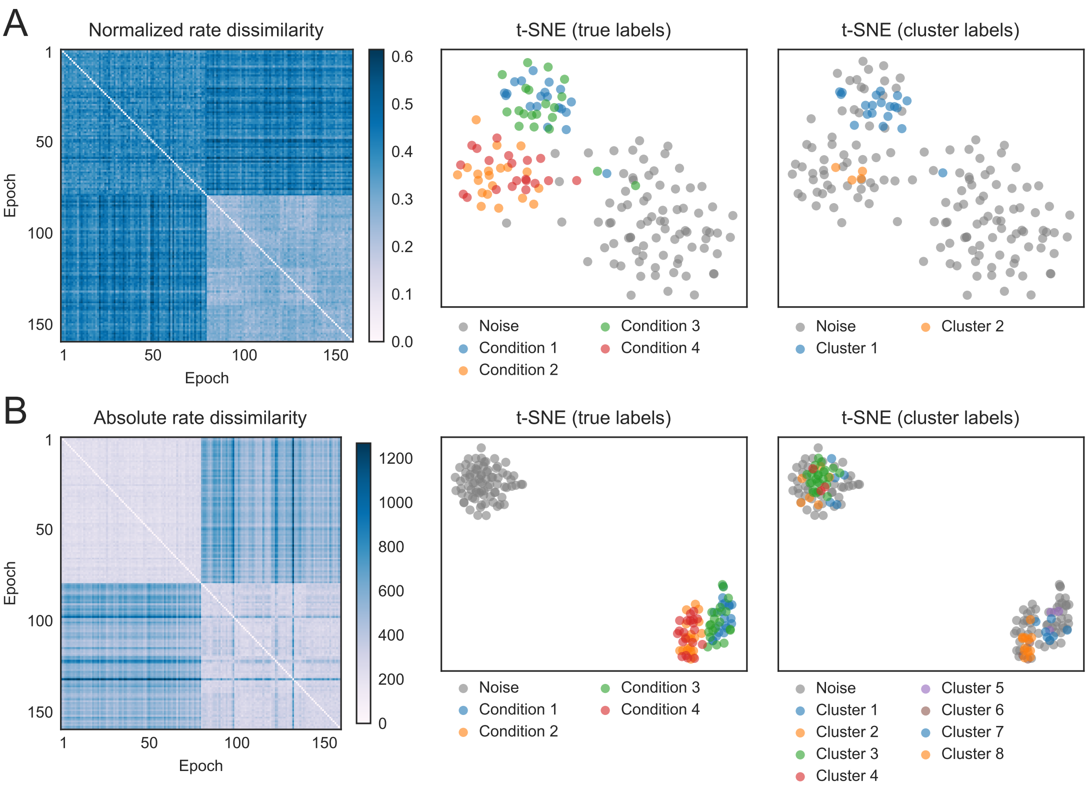

Figure S10: Application to neuronal data, matching Figure 8. For each multi-unit, we computed the spike count in the same temporal window as used for the SPOTDisClust clustering, denoted r_{ik} (epoch k, unit i). In (A), we then constructed a normalized population vector as $r'_{ik} = r_{ik}/\sum_i r_{ik}$ for each multi-unit i. We then constructed all pairwise distances between epochs k and m as the L1-norm among these normalized population vectors, $D^{rate}_{km} = \frac{1}{N}\sum_{i=1}^{N}|r'_{ik} - r'_{im}|$. Based on these pairwise distances, we then performed low dimensional t-SNE embedding and HDBSCAN clustering. Shown are the distance matrix, as well as the t-SNE with true labels and cluster labels. The clustering and low-dimensional embedding is unable to separate out all four stimulus directions. In (B), we defined the pairwise distances as the L1-norm on the absolute spike counts, i.e. $D^{rate}_{km} = \frac{1}{N}\sum_{i=1}^{N}|r_{ik} - r_{im}|$, and then performed t-SNE and HDBSCAN clustering. Like in (A), this clustering procedure fails to separate all four stimulus directions.
