# Supplementary material for: Unsupervised clustering of temporal patterns in high-dimensional neuronal ensembles using a novel dissimilarity measure
Source: PLoS Comput Biol. 2018 Jul 6;14(7):e1006283. doi: 10.1371/journal.pcbi.1006283 (PMC6051652; doi:10.1371/journal.pcbi.1006283)
Supplement: S11 Fig — The parameter settings for the patterns underlying this figure are equivalent to Fig 1. Each pattern has a length of 300 samples, and is embedded in a larger window starting from -300 samples to +300 samples, with homogeneous noise surrounding the pattern on the left and right. The onset of the pattern is -150 samples plus some random offset Δtw. For each epoch realization, the value of Δtw was randomly chosen with uniform probability from an interval determined by the maximum window offset (max offset of 100 meant that Δtw ∈ [−100, 100]). A value of Δtw = −50 then meant that the sequence started at -200 samples and ended at 100 samples. For the clustering, we then assume that the sequence duration is unknown. We select a window ranging from −Tw/2 to +Tw/2 samples of length Tw. In case of Tw = 300 and no offset (Δtw = 0), the generated data matches the simulation of 1. Clustering performance was measured relative to ground-truth (ARI) and with an unsupervised performance measure, Silhouette (see Methods). Clustering performance decreased as the maximum window offset increased, due to the inclusion of noise spikes around the spike pattern. Clustering performance peaked around Tw = 300 samples for both ARI and Silhouette as Tw. indicating that we can determine the “optimal” window length in an unsupervised manner when the sequence duration is unknown. (PDF) [file pcbi.1006283.s011.pdf]

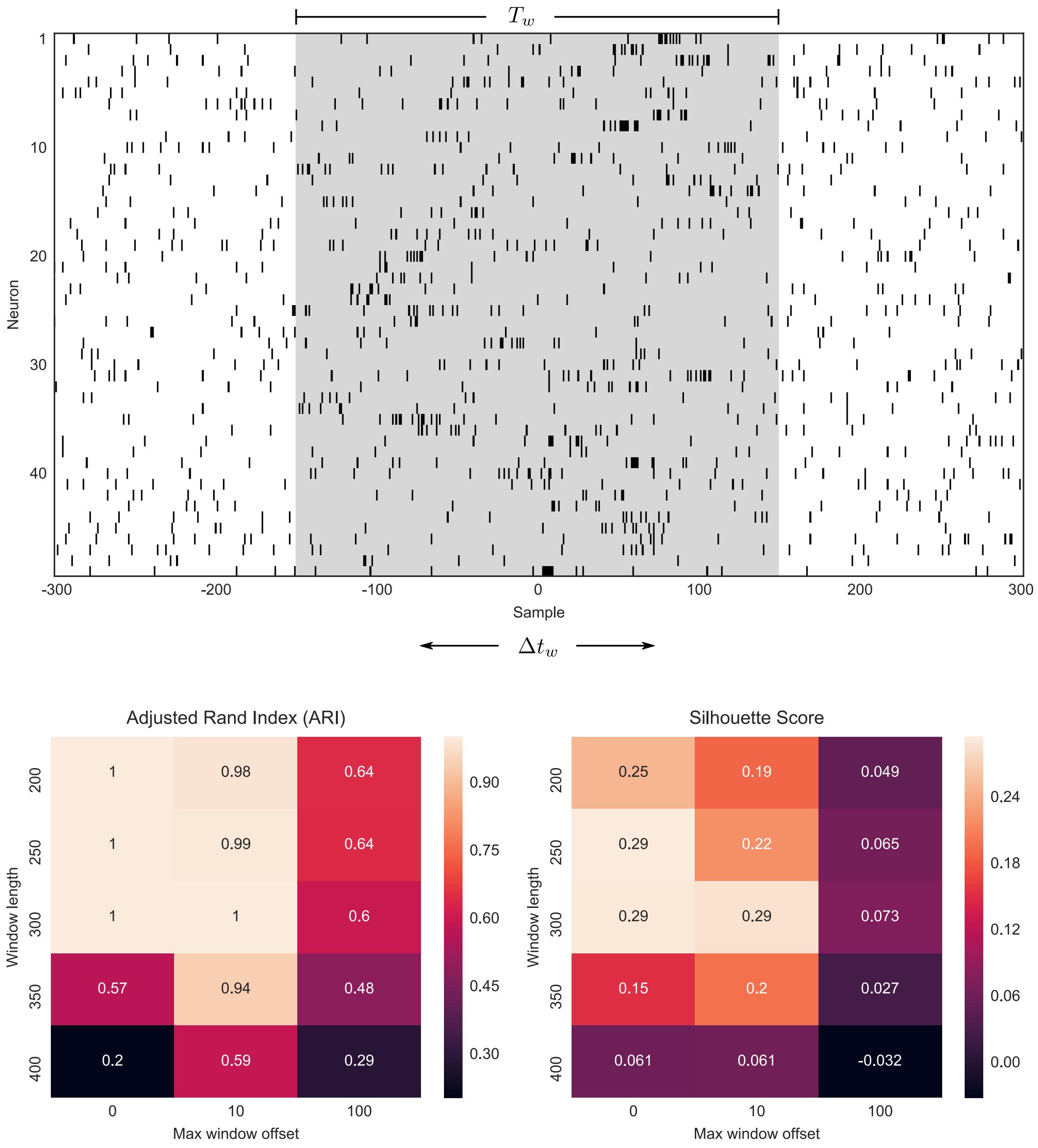

Figure S11: Dependence of clustering performance on chosen window length and temporal jitter of spike pattern onset. The parameter settings for the patterns underlying this figure are equivalent to Figure 1. Each pattern has a length of 300 samples, and is embedded in a larger window starting from -300 samples to +300 samples, with homogeneous noise surrounding the pattern on the left and right. The onset of the pattern is -150 samples plus some random offset Δt_w. For each epoch realization, the value of Δt_w was randomly chosen with uniform probability from an interval determined by the maximum window offset (max offset of 100 meant that $\Delta t_w \in [-100, 100]$). A value of $\Delta t_w = -50$ then meant that the sequence started at -200 samples and ended at 100 samples. For the clustering, we then assume that the sequence duration is unknown. We select a window ranging from $-T_w/2$ to $+T_w/2$ samples of length T_w. In case of $T_w = 300$ and no offset ($\Delta t_w = 0$), the generated data matches the simulation of Figure 1. Clustering performance was measured relative to ground-truth (ARI) and with an unsupervised performance measure, Silhouette (see Methods). Clustering performance decreased as the maximum window offset increased, due to the inclusion of noise spikes around the spike pattern. Clustering performance peaked around $T_w = 300$ samples for both ARI and Silhouette as T_w. indicating that we can determine the "optimal" window length in an unsupervised manner when the sequence duration is unknown.
