# Supplementary material for: Unsupervised clustering of temporal patterns in high-dimensional neuronal ensembles using a novel dissimilarity measure
Source: PLoS Comput Biol. 2018 Jul 6;14(7):e1006283. doi: 10.1371/journal.pcbi.1006283 (PMC6051652; doi:10.1371/journal.pcbi.1006283)
Supplement: S12 Fig — We repeated the clustering with different window length selections. Windows were centered on 180 ms, the center of the 360 ms stimulation period. Clustering performance, measured both with a ground-truth performance measure (ARI) and an unsupervised cluster quality score (Silhouette), peaked at the window length of 360 ms. Large window lengths lead to an inclusion of baseline firing, decreasing clustering performance, whereas smaller window lengths also deteriorated performance. ARI and Silhouette scores showed a tight correspondence, showing the feasibility of using Silhouette score to “optimize” the window length for sequence detection. (PDF) [file pcbi.1006283.s012.pdf]

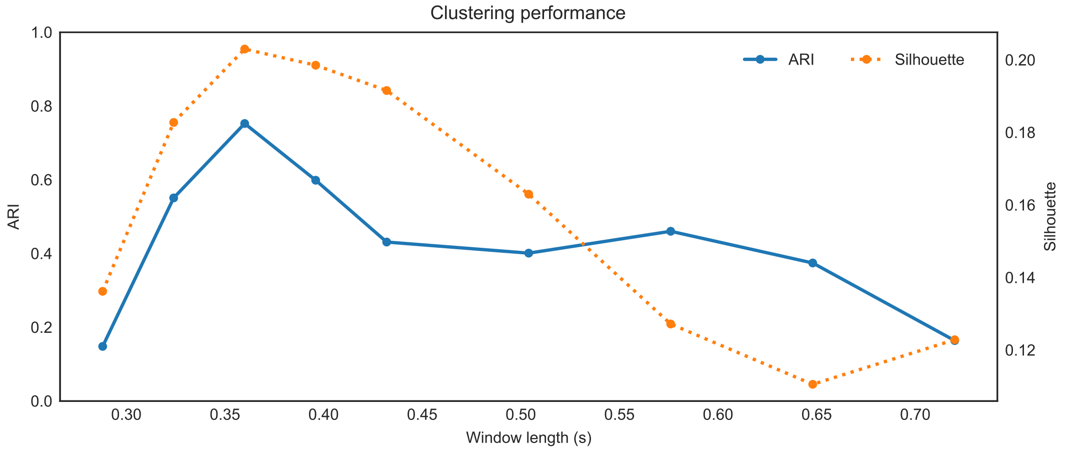

Figure S12: Application to neuronal data of window length selection, matching Figure 8. We repeated the clustering with different window length selections. Windows were centered on 180 ms, the center of the 360 ms stimulation period. Clustering performance, measured both with a ground-truth performance measure (ARI) and an unsupervised cluster quality score (Silhouette), peaked at the window length of 360 ms. Large window lengths lead to an inclusion of baseline firing, decreasing clustering performance, whereas smaller window lengths also deteriorated performance. ARI and Silhouette scores showed a tight correspondence, showing the feasibility of using Silhouette score to "optimize" the window length for sequence detection.
